# The Effect of Amlodipine and Sildenafil on the NT-ProBNP Level of Patients with COPD-Induced Pulmonary Hypertension 

**Published:** 2014

**Authors:** Babak Sharif-kashani, Ali Hamraghani, Jamshid Salamzadeh, Mohammad Abbasi Nazari, Majid Malekmohammad, Neda Behzadnia, Fanak Fahimi

**Affiliations:** a *Lung Transplantation Research Center, NRITLD, Masih Daneshvari Hospital, Shahid Beheshti University of Medical Sciences, Tehran, Iran.*; b *Clinical Pharmacy Department, School of Pharmacy, Shahid Beheshti University of Medical Sciences, Tehran, Iran. *; c *Tracheal Diseases Research Center, National Research Institute of Tuberculosis and Lung Diseases (NRITLD), Shahid Beheshti University of Medical Sciences, Tehran, Iran.*; d *Chronic Respiratory Disease Research Center, NRITLD, Masih Daneshvari Hospital, Shahid Beheshti University of Medical Sciences, Tehran, Iran. *

## Abstract

Pulmonary hypertension (PH) is an important cause of heart failure in chronic obstructive pulmonary disease (COPD). The pro brain natriuretic peptide N-terminal (NT-proBNP) has been suggested as a noninvasive marker to evaluate ventricular function. However, there is no evidence to support the use of NT-proBNP in monitoring the benefits of vasodilators in COPD induced PH. Thus, we used NT-proBNP as a biomarker to evaluate the effect of oral vasodilators on cardiac function in COPD-induced PH.

Forty clinically-stable PH patients were enrolled with history of COPD, normal left ventricular ejection-fraction (LVEF), right ventricular systolic pressure (RVSP) > 45 mmHg and baseline blood NT-proBNP levels >100 pg/mL. Patients were randomized into two groups, one group received sildenafil and second group were given amlodipine for two weeks. NT-proBNP and systolic pulmonary arterial pressure (systolic PA-pressure) were measured at the beginning and the end of study.

Mean NT-proBNP level in the first group was 1297 ± 912 pg/mL before therapy and 554 ± 5 pg/mL after two weeks drug therapy, respectively. Similarly, in second group NT-proBNP level was 1657 ± 989 pg/mL and 646 ± 5 pg/mL before and after treatment. Amlodipine or sildenafil significantly reduced NT-proBNP levels in COPD-induced PH patients (p < 0.05).

Our study shows that amlodipine and sildenafil have a similar effect on NT-proBNP levels. In both groups NT- proBNP levels were significantly reduced after treatment. Therefore, our findings support the potential benefits of treatment with vasodilators in COPD induced PH.

Pulmonary hypertension, Chronic obstructive pulmonary disease, NT-proBNP, Amlodipine, Sildenafil

## Introduction

Chronic obstructive pulmonary disease (COPD) is a leading cause of morbidity and mortality worldwide with an increasing prevalence (1). Pulmonary hypertension (PH) is a condition characterized by increased resistance to pulmonary blood flow and leads to right heart failure. In fact, previous large trials have mentioned that the most frequent cause of death in COPD patients is cardiac rather than respiratory complication (2,^3^). PH and corpulmonale are important causes of death and poor prognosis in COPD (4, 5).

Currently, there is no specific therapy for PH associated with COPD. Long-term Oxygen administration has been shown partially to reduce the progression of PH in COPD. Despite this treatment, pulmonary arterial pressure (PAP) not often returns to normal values and the structural abnormalities of pulmonary vessels remain untouched (6). Specific therapies such as vasodilators have been associated with inconsistent results and safety and efficacy are not well established (7). Calcium channel blockers (CCBs) were the first class of drugs shown to benefit patients with PH (8); Then Phosphodiesterase inhibitors (PDEI) have been tested in small numbers. The study was reported pulmonary hemodynamics improvement after the sildenafil usage (9).

Natriuretic peptides are hormones secreted by heart in response to hemodynamic stress and structural abnormalities of the heart. Moreover, circulating N-terminal of pro brain natriuretic peptide (NT-proBNP) concentration has recently been shown to correlate well with survival and echocardiography-derived measures of right ventricular (RV) function in PH (10). In addition, the close correlation with the hemodynamic data and the acute variation during vasodilator therapy suggest that NT-proBNP may also be used as prognostic and treatment marker. It would be clinically useful not only for monitoring the respond of vasodilators, but also to evaluate the behavior of the cardiovascular system with the use of the available therapies (11). Previous study by Nagaya et al. have shown that circulating BNP may also serve as a noninvasive marker for the efficacy of therapy in chronic thromboembolic pulmonary hypertension (CTEPH) patients (12).

We assessed NT-proBNP levels along with systolic pulmonary arterial pressure (systolic PA-pressure) to monitor RV function in COPD-induced PH patients. Also for the first time, response to oral vasodilators (sildenafil and amlodipine) was evaluated by NT-proBNP in these patients.

## Experimental

This prospective, randomized, open-label parallel group study was carried out at the National Research Institute of Tuberculosis and Lung Disease (NRITLD), Masih Daneshvari Hospital, Tehran, Iran, between May 2008 and July 2009.

Inclusion criteria were diagnosis of COPD patients without exacerbation or hospital admission in the past two months, 18 to 75 years of age, right ventricular systolic pressure (RVSP) greater than 45 mmHg and baseline blood NT-proBNP levels above 100 pg/mL. Patients data including past medical history, blood tests results and transthoracic echocardiogram. Patients with coexisting conditions likely to elevate NT-proBNP levels, such as pulmonary embolism (13), ischemic heart disease (14), myocardial infarction (15), left ventricular systolic dysfunction (LVSD) (ejection fraction <40 %) (16), systemic hypertension (blood pressure >150/90 mmHg) (17), left-sided valvular heart disease (18), renal impairment (19), diabetes mellitus (20), and anemia (21) were excluded from the study. Also patients were excluded if they had severe concomitant disease (infection, cancer), or used medications which may change NT-proBNP levels (other vasodilators). Other exclusion criteria were as follows: unwillingness of the patient to continue, acute exacerbation of COPD during the study period, the development of any serious side effects significantly affecting quality of life (persistent severe pedal edema or headache). At the time of the study, no patient had received any disease targeted therapy (i.e. prostacyclin analogues, endothelin antagonists, phosphodiesterase inhibitors or calcium-channel blockers) for PH.

Treatment started at the time of randomization (baseline) for two weeks. In group (A) patients received sildenafil

25-50 mg two times daily and in group (B) patients took amlodipine 2.5-7.5 mg once daily. These dosage ranges of medication were decided by cardiologist (BSK) based on blood pressure and patient tolerance. NT-proBNP and systolic PA-pressure levels were measured before and after the two weeks of drug administration.

*Diagnostic tests*

Patients were evaluated by at least two physician board-certified internal medicine specialists during outpatient visits. The diagnosis of COPD was based on clinical history, physical examination and spirometric criteria according to Global Initiative for Chronic Obstructive Lung Disease (GOLD) guidelines (22). Then stable COPD patients underwent echocardiography.

*Echocardiography*

Echocardiographic studies were performed by a cardiologist, experienced in evaluation of pulmonary heart disease and parameters from Doppler analysis, M-mode and two-dimensional trans-thoracic echocardiographies were used. Furthermore, we measured the peak velocity of the tricuspid regurgitant signal by continuous wave doppler and calculated the RVSP with the modified Bernoulli equation, which is believed to reflect systolic PA-pressure in the absence of RV outflow tract obstruction. Theoretically, calculation of mPAP from systolic PA-pressure (PASP) is possible, {mPAP = (0.61×PASP) + 2 mmHg} (23).

*NT-proBNP measurement*

After the echocardiography, eligible patients selected to measure blood NT-proBNP levels. Heparinized venous whole blood samples (without centrifugation or other additional preparation) from each patient were analyzed for NT-proBNP, using a validated, commercially available immunoassay (Cobas 232 h, Roche Cardiac Diagnostics), using established methodology (24). Results are given in pg/mL. All physicians directly involved in the patient care were blinded to NT-proBNP values.

*Data analysis*

Data were expressed as mean ± SD and median where appropriate, and analyzed using SPSS 16.0 for Windows. All variables were tested for normal distribution with the Kolmogorov-Smirnov test. Dependent of their distribution, Wilcoxon’s test was used to determine if there were significant differences among NT-proBNP levels after two weeks drug therapy in the two different groups (paired analysis) and comparison between sildenafil group and amlodipine group was performed using the Mann-Whitney U-test (parallel analysis).

Spearman›s correlation analyses were performed to investigate significant correlations and Kruskal-Wallis test was used to determine if there were significant differences among baseline values of two different groups. A value of p < 0.05 was considered significant.

The study was approved by the medical ethics committee of the Shahid Beheshti University of Medical Science

(Tehran, Iran) and written informed consent was obtained from all patients.

## Results

**Figure 1:**
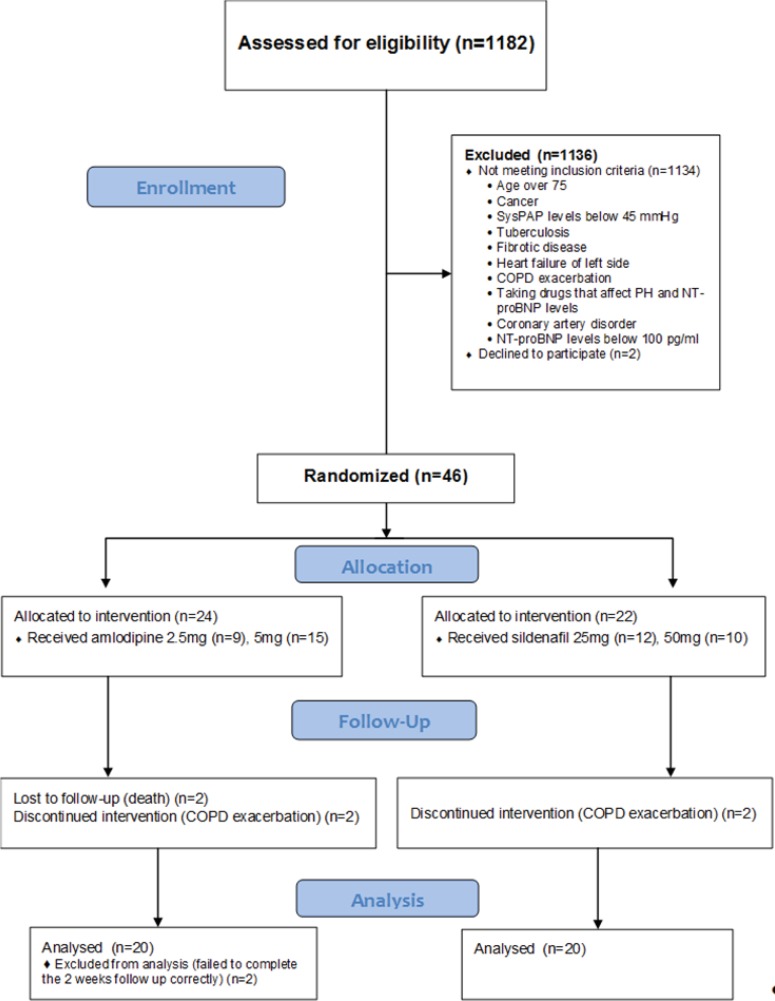
Flow chart of the patients

A total of 1182 patients, with the diagnosis of COPD were screened for enrolment. Forty six patients were eligible and underwent randomization. Six patients withdrew from the study. Two patients in each group discontinued because of acute COPD exacerbation. Moreover, two patients in group (B) excluded because their follow up session was taken more than two weeks. Finally, forty clinically-stable COPD patients with PH completed the follow up correctly (Figure 1).

*Baseline Characteristics*

The mean ± SD age of the studied population was 61.6 ± 7.8 with a range of 48-75 years and the majority (77%) of patients was male. A comparison of baseline parameters between two groups demonstrated no significant difference in age, gender, smoking, systolic PA pressure and NT-proBNP (Table 1), reflecting randomized allocation.

The baseline clinical characteristics of study population are summarized in Table 1.

**Table 1:** Baseline clinical characteristics of the subjects

**Parameters**	**Subjects(n=40)**	**Group A (Sildenafil) (n=20)**	**Group B (Amlodipine)(n=20)**	**Group A vs. B(p-value)**
Age, mean ± SD	61.6 ± 7.8	60.2 ± 6.9	63.0 ± 8.5	0.26
Gender (M:F)	31:9	16:4	15:5	1.00
Smoking (N:Y)	17:23	9:11	8:12	0.75
Systolic PAP (mmHg)*	60.5 ± 12.3	58.0 ± 11.8	63.0 ± 12.5	0.15
NT – proBNP(pg/mL)*	1477.5 ± 957.0	1297.5 ± 912.8	1657.4 ± 989.1	0.23

*mean ± SD

*NT-proBNP and Oral vasodilators effects*

**Figure 2:**
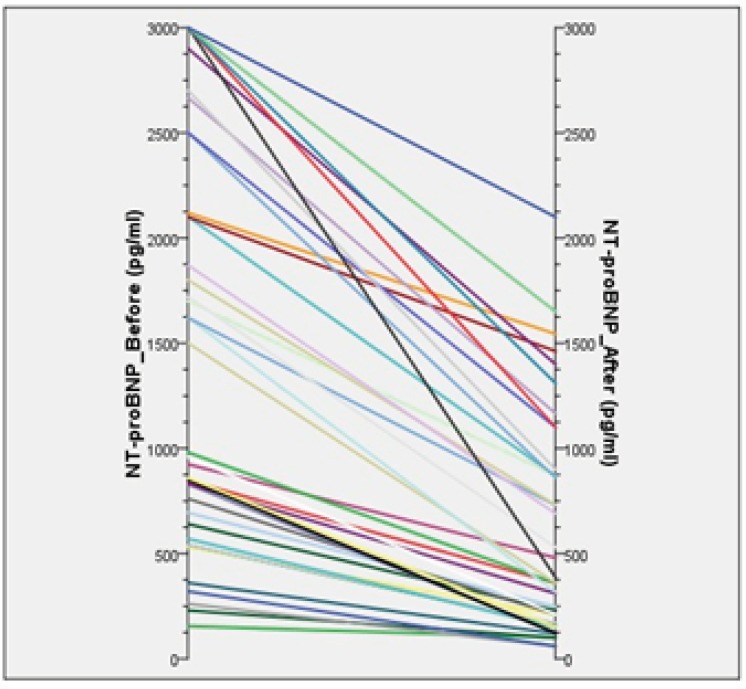
Parallel diagram of the changes

Both sildenafil and amlodipine reduced NT-proBNP levels significantly in patients with COPD-induced PH after two weeks of treatment (Figure 2). Also, there was a significant reduction in systolic PA-pressure levels along with NT-proBNP.

In group A which sildenafil was given, plasma NT-proBNP concentration was reduced after two weeks treatment significantly. Median of NT-proBNP value was 942 pg/mL and 273 pg/mL (p < 0.001) at baseline and after intervention, respectively. Like NT-proBNPs a significant decrease was identified in systolic PA-pressure (Table 2).

**Table 2:** Baseline and after treatment values

	**Baseline**	**After treatment**	**p** ***-*** **value**	**Baseline**	**After treatment**	**p** ***-*** **value**
Sildenafil (n=20)			0.001			0.001
Median Interquartile range	942 547-1827	273 130-835		55 50-67.5	45 40-50	
Amlodipine (n=20)			0.001			0.001
Median Interquartile range	1760 777-2624	458 219-1050		62.5 55-70	47.5 45-53.75	

In group B that patients treated with amlodipine, there was a significant reduction in plasma NT-proBNP concentration after two weeks drug therapy was seen. The baseline median value was 1760 pg/mL before treatment and 458 pg/mL after treatment (p < 0.001). Similar to NT-proBNP, the fall in systolic PA-pressure was statistically significant in both group (Table 2).

The NT-proBNP level of patients in both groups was significantly decreased after receiving the study medications. However, comparison between group A and B indicated no significant difference between sildenafil and amlodipine in lowering the NT-proBNP levels (p = 0.185) (Table 3).

**Table 3:** The outcome measures comparison between sildenafil and amlodipine

**Median (IQR) reduction**	**Sildenafil**	**Amlodipine**	**p-value**
NT – proBNP level (pg/mL)	717	890	0.181
Systolic PAP level (mmHg)	10	15	0.164

*Relationship between change in NT-proBNP and echocardiography findings*

There was a significant correlation between pre-treatment baseline NT-proBNP levels and baseline systolic PA- pressure (r= 0.746, p < 0.001). Also the correlation between estimated NT-proBNP and systolic PA-pressure levels after two weeks treatment was significant (r = 0.727, p < 0.001).

*Mortality*

The high plasma NT-proBNP concentration (>3000 pg/mL) was the reason for exclusion of four patients (two patient in group A and two patient in group B). The two patients in group B died in the hospital during the period less than three weeks.

## Discussion

Results from our study pointed, NT-proBNP and systolic PA-pressure levels in COPD induced PH patients after a two-week drug therapy with sildenafil or amlodipine were reduced statistically significant. In fact, decreasing NT- proBNP levels may be an indicator of the efficacy of treatment with amlodipine or sildenafil on improving RV function in COPD induced PH. NT-proBNP level increases due to pulmonary hypertension and RV overload (25). Lowering NT-proBNP and systolic PA-pressure levels indicate these vasodilators may reduce PAP and cardiac load in COPD patients with PH and further may inhibit the synthesis and secretion of endothelin and pulmonary vascular remodeling. Therefore, our findings support a working hypothesis for the benefits of treatment with vasodilators in COPD induced PH.

To compare the effect of these drugs in reducing NT-proBNP levels, no significant difference was observed between sildenafil and amlodipine.

There are relatively few studies investigating the alteration of NT-proBNP levels with respect to monitor the treatment in PH patients and no study to date has directly evaluated the effect of oral vasodilators in COPD induced PH patient’s RV function with NT-proBNP. It has been shown by Fijalkowska *et al*. (10) that NT-proBNP is related to right heart morphology and dysfunction as assessed by echocardiography and right heart catheterization in PH patients. Ishii (25) also showed that plasma BNP concentration closely correlated with the mean pulmonary arterial pressure and pulmonary vascular resistance in patients with chronic respiratory diseases. Previous studies by Nagaya *et al. *(12) in Chronic Thromboembolic Pulmonary Hypertension (CTEPH) patients and Wilkins *et al*. (26) who treated patients with sildenafil and bosentan have shown that circulating BNP concentration correlates with the response to therapy in PH patients.

Consistent with the previous studies (Ishii et al. (25), Fijalkowska et al. (10)) our present results showed that plasma NT-proBNP levels and systolic PA-pressure were both significantly decreased after treatment. We hypothesized NT-proBNP plays an important role in the pathophysiology of pulmonary hypertension and it could be used as an indicator of disease severity to compare the effect of vasodilators in COPD induced PH.

Our study has some limitations, as it is the first in this respect. Right heart catheterization was not done in our center at the time of study. However, echocardiography was performed for all patients. Doppler echocardiography allows an estimation of the pulmonary arterial pressure and provides information about right and left ventricular function. Another potential limitation of this analysis was the lack of a placebo control in the open-label portion of the study while it might have been interesting to learn the extent of difference between the vasodilator-treated and placebo- treated patients. Also, more evaluation of the safety, efficacy and optimal dosing of these medications is needed prior to the routine use of these therapies in the management of COPD induced PH patients. Before considering a vasodilator therapy as a possible therapeutic strategy in COPD induced PH patients with high plasma NT-proBNP levels, additional data should be obtained from studies enrolling large number of patients but since PH is a relatively rare disease, it may not be feasible to power a clinical trial with a sufficient number of patients to effectively use these treatments. In order to assess the effect of sildenafil and amlodipine on quality of life and exercise capacity, 6- min walk distance (6MWD) test and a cardiopulmonary exercise test (CPET) should be performed.
